# Supplementary figures and images for: CFSAN SNP Pipeline 2 (CSP2): a pipeline for fast and accurate SNP distance estimation from bacterial genome assemblies
Source: PeerJ Comput Sci. 2025 Jul 9;11:e2878. doi: 10.7717/peerj-cs.2878 (PMC12453658; doi:10.7717/peerj-cs.2878)

**A**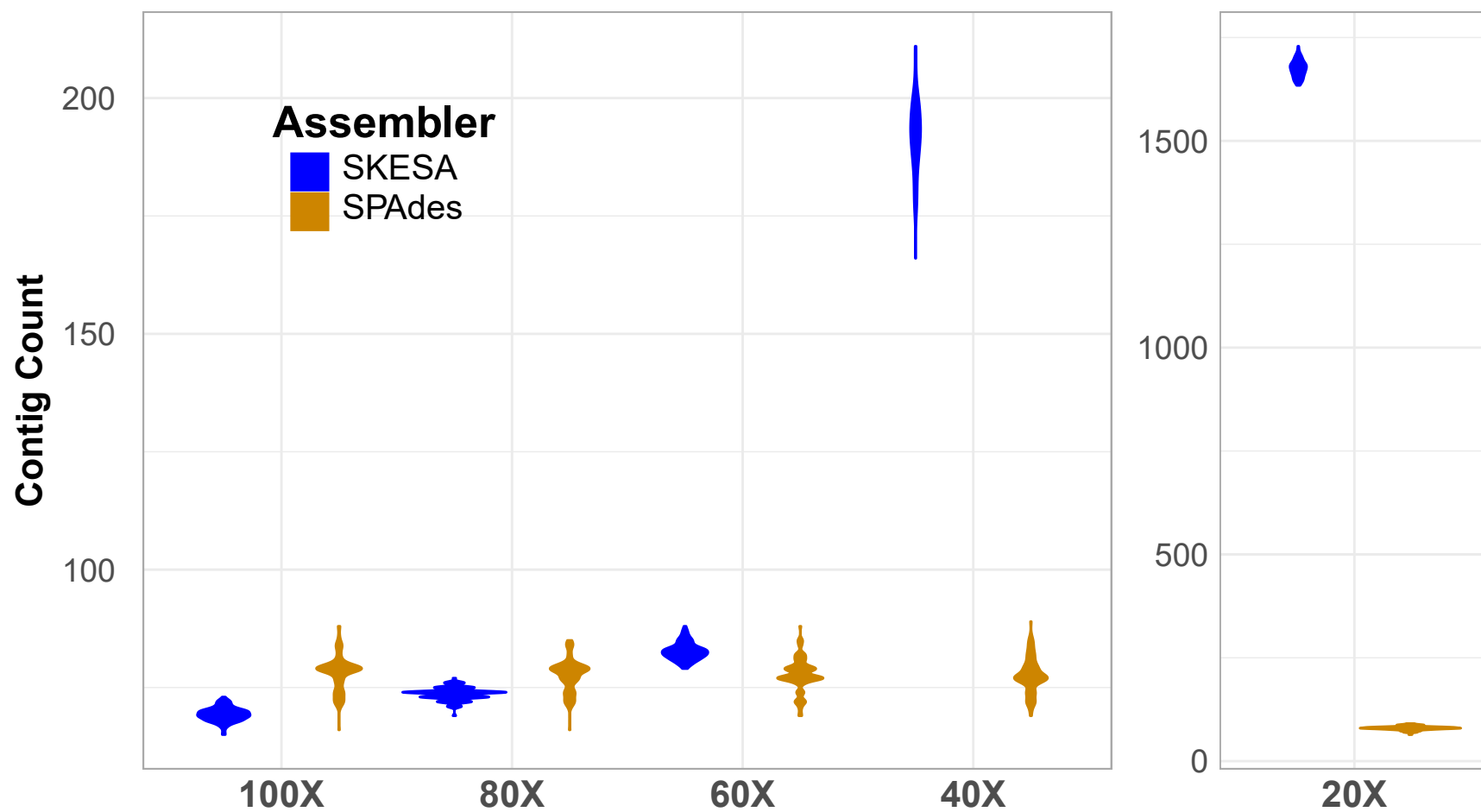**B**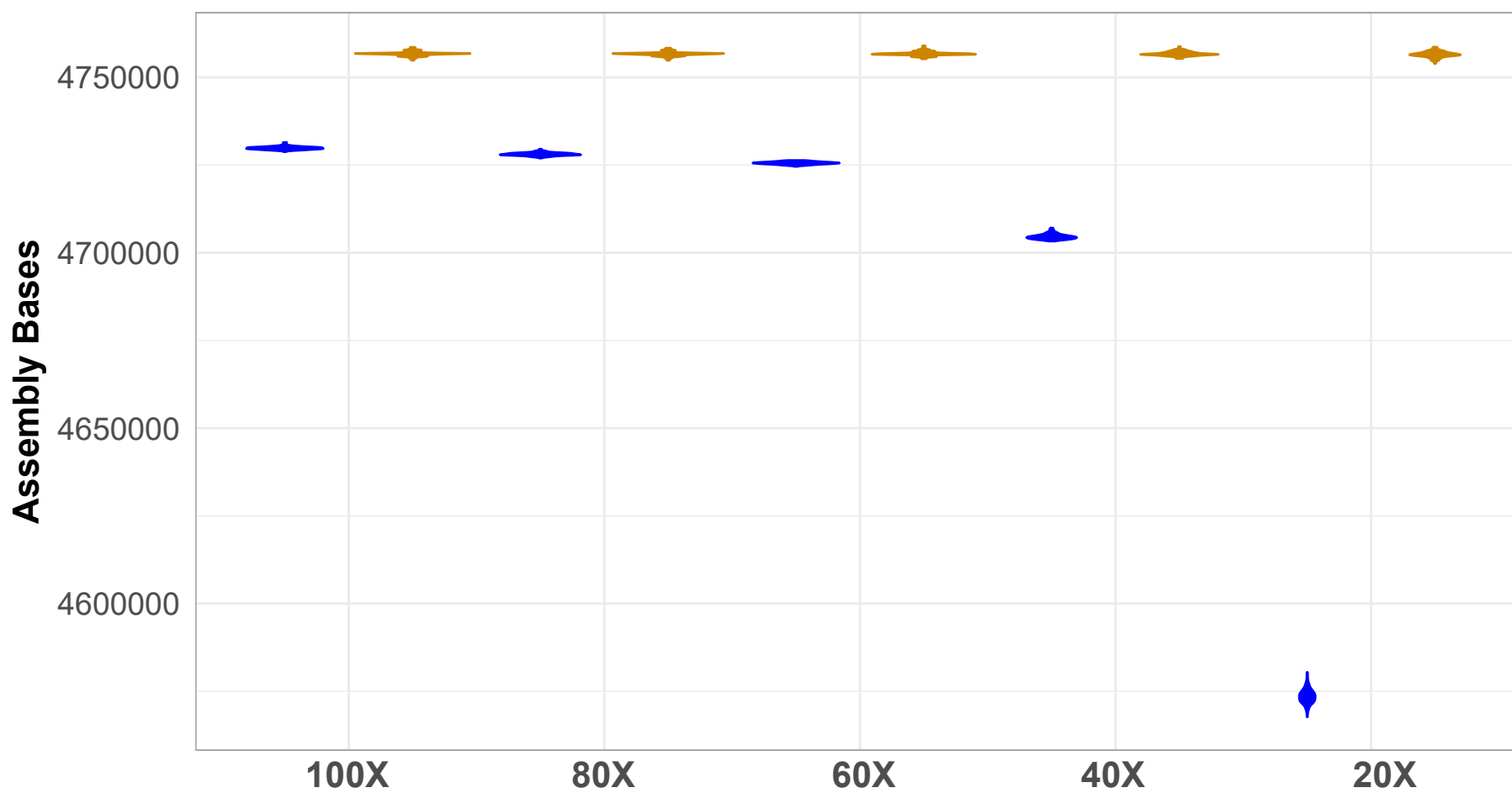

Supplement: Supplemental Information 1 — (A) Contig counts for SPAdes and SKESA assemblies from different starting read depths. (B) Assembly sizes for SPAdes and SKESA assemblies. [file peerj-cs-11-2878-s001.pdf]

**A**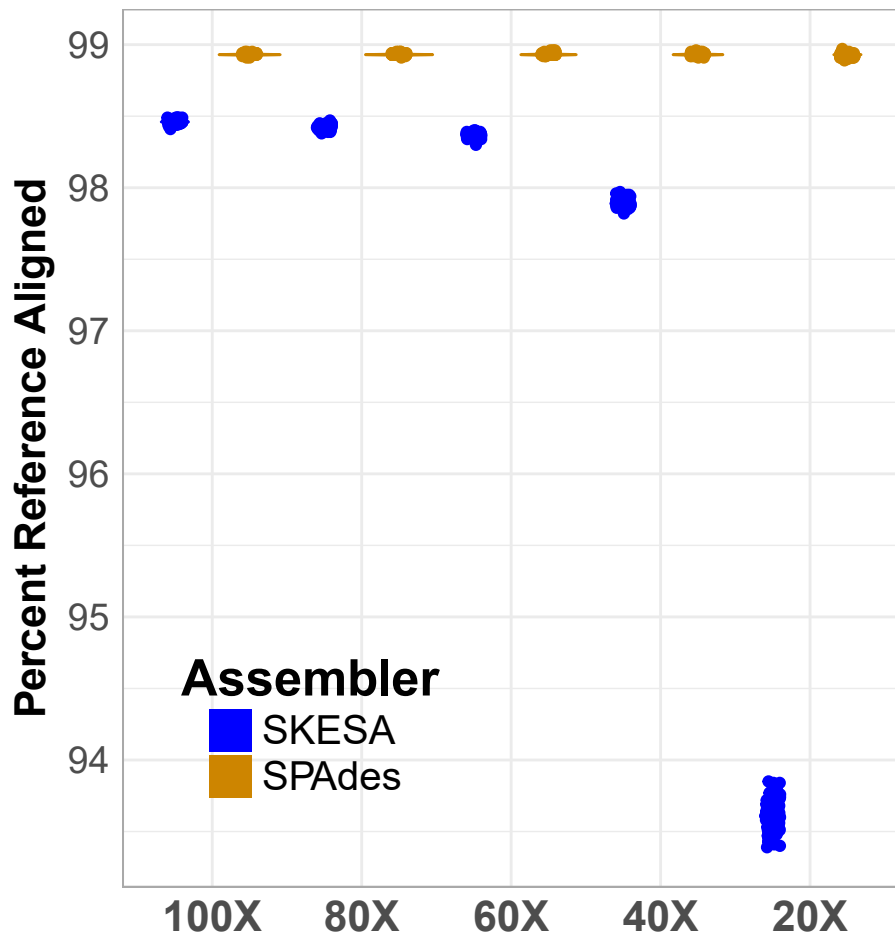**B**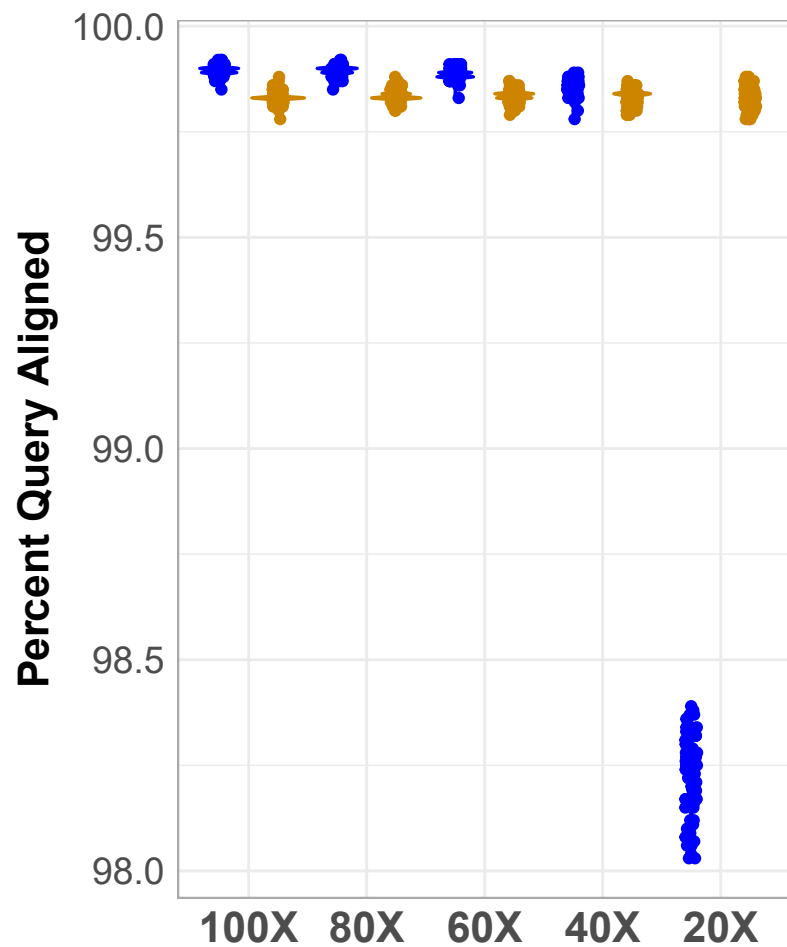

Supplement: Supplemental Information 2 — (A) Percent reference genome alignment (A) and (B) percent query alignment for SPAdes and SKESA assemblies across depths. 20X SKESA assemblies were especially fragmented. [file peerj-cs-11-2878-s002.pdf]

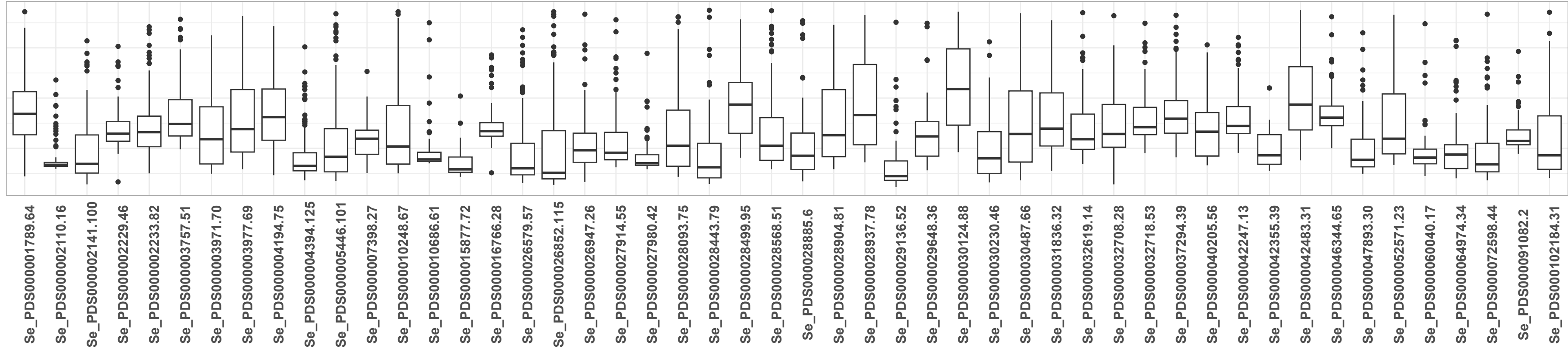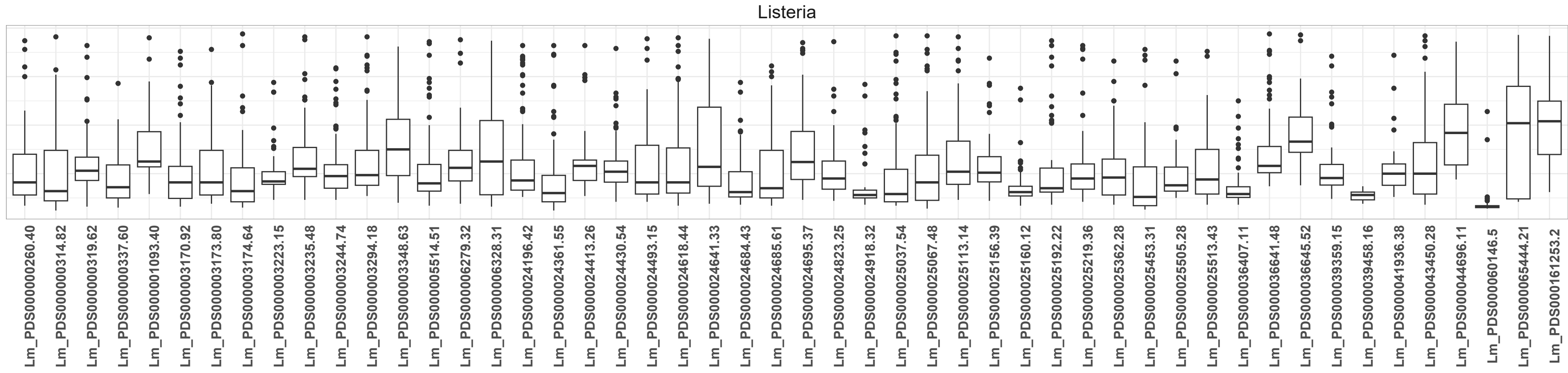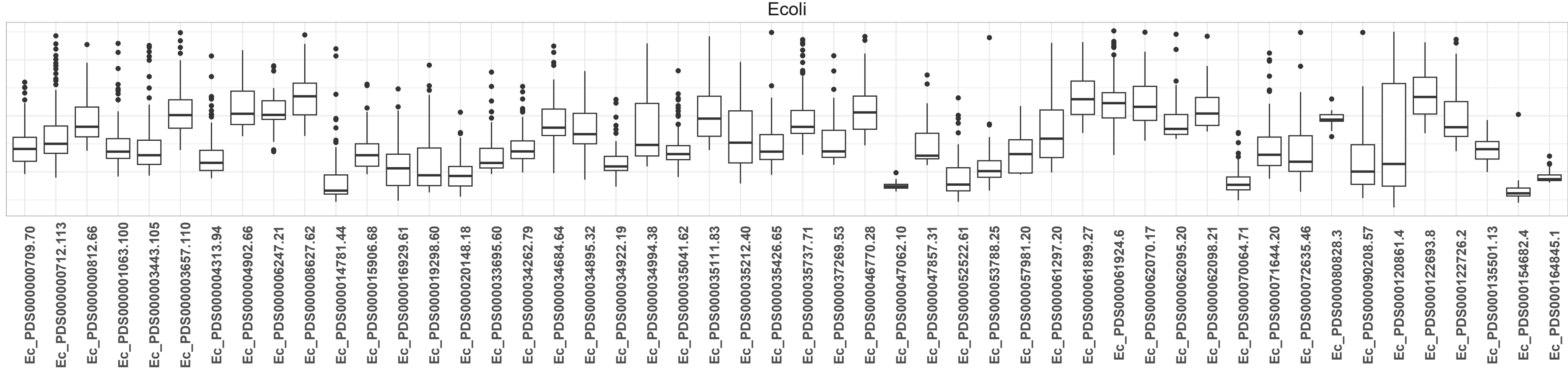

Supplement: Supplemental Information 3 — Contig counts for each cluster in the analysis. [file peerj-cs-11-2878-s003.pdf]
